# Complete form of pachydermoperiostosis^☆^^☆☆^

**DOI:** 10.1016/j.abd.2019.04.009

**Published:** 2019-12-18

**Authors:** Mônica Larissa Padilha Honório, Guilherme Holanda Bezerra, Vivianne Lira da Câmara Costa

**Affiliations:** Dermatology Service, Hospital Universit´rio Onofre Lopes, Universidade Federal do Rio Grande do Norte, Natal, RN, Brazil

**Keywords:** Adolescent health, Heredity, Osteoarthropathy, Primary hypertrophic

## Abstract

Pachydermoperiostosis (PDP) or primary hypertrophic osteoarthropathy (PHO) is a rare hereditary disease characterized by digital clubbing, pachydermia, and periostosis. Its pathogenesis is uncertain and the diagnosis is based on clinical and radiological data. A complete form of the syndrome is reported in a male patient with disease onset in adolescence, with compatible clinical and radiological findings, presenting the three cardinal findings as well as other associated manifestations, such as hyperhidrosis and acne.

## Introduction

Pachydermoperiostosis (PDP) or primary hypertrophic osteoarthropathy (PHO) is a rare hereditary disease that was first described in 1868.^1^ Its actual incidence is unknown,^2^ but as evidenced by the MEDLINE search, a total of 286 reported cases were found. Adolescent males are predominantly affected, with male-to-female ratio of approximately 7:1.^3^

Hypertrophic osteoarthropathy (HOA) is divided into primary and secondary forms. PDP, the primary form, accounts for 3–5% of all cases of HOA. Secondary HOA, also called pulmonary HOA, is associated with underlying cardiopulmonary diseases and malignancies. The diagnosis of PDP is established with clinical and radiological data.^1^, ^2^

This report details a case of pachydermoperiostosis in a young male patient.

## Case Report

**Figure 1:**
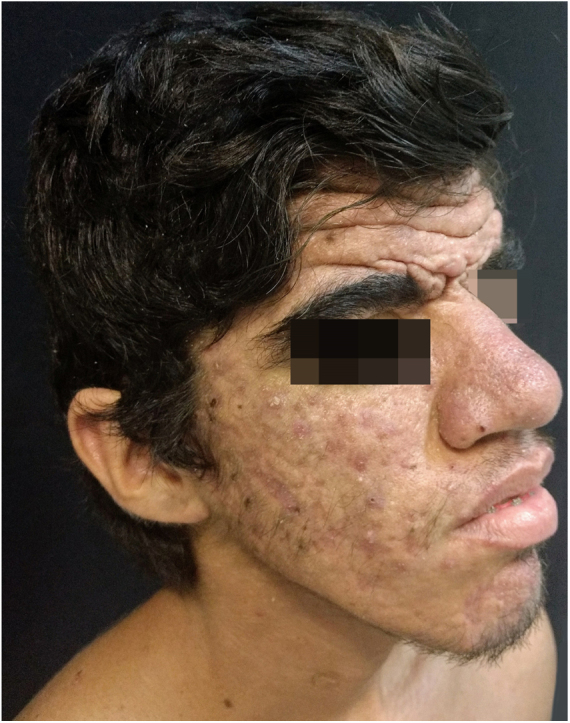
Erythematous papules and pustules on face, skin thickening, and accentuation of facial furrows with cutaneous gyrata (cutis verticis gyrata) on the forehead.

**Figure 2:**
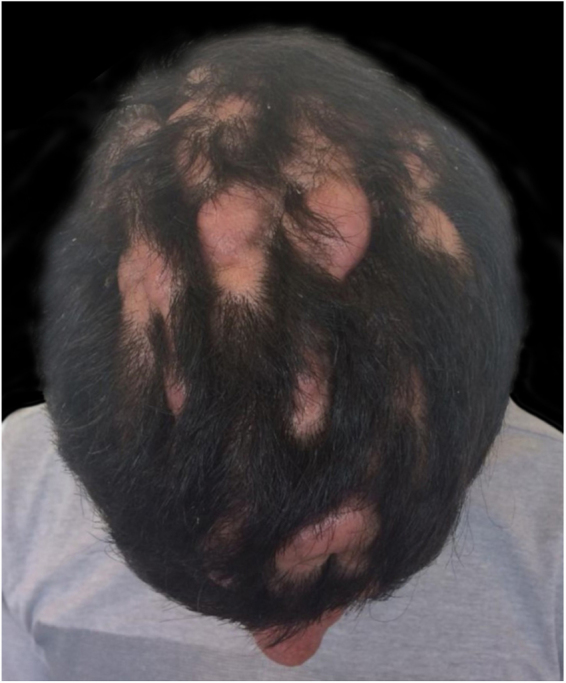
Scalp with cutaneous convolutions and soft nodules with alopecia on their surfaces.

**Figure 3:**
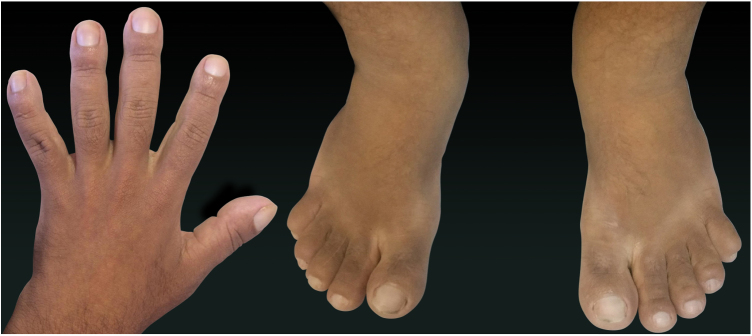
Fingers and toes showing volume increase and digital clubbing.

**Figure 4:**
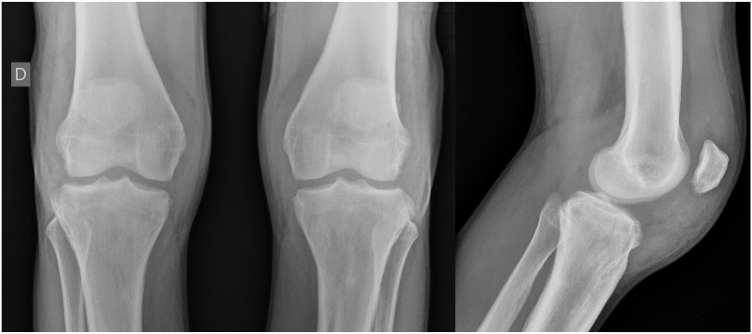
Benign hypertrophic periosteal reaction in metaphyses and epiphyses of the femur, tibia, and fibula bilaterally, with enlargement of the bones due to hyperostosis. Densification and increase of the thickness soft parts adjacent to the knee, especially in the anterior and lateral faces.

A 24-year-old male patient reported the following since his teens: skin thickening on the face and scalp, as well as volume increase of the hands and feet. He also reported acne since adolescence. The patient denied any previous family history. Physical examination revealed erythematous follicular papules and pustules on face, cutaneous thickening, and accentuation on facial furrows with cutis verticis gyrata on the forehead (Fig. 1) and scalp, where there were soft nodules with alopecia on their surfaces, compatible with abscessing folliculitis (Fig. 2). The hands and feet showed volume increase and digital clubbing (Fig. 3), as well as increased sweating on the hands and feet (hyperhidrosis). The radiographs of the hands, fists, and knees showed a benign hypertrophic periosteal reaction with bone enlargement by hyperostosis, as well as densification and increase of the thickness of adjacent soft tissues (Fig. 4). The insulin-like growth factor-1 (IGF-1) levels were normal. Based on clinical characteristics (pachydermia, digital clubbing, and periostosis) the complete form of pachydermoperiostosis was diagnosed.

## Discussion

PDP is a genetic syndrome characterized by three main characteristics: digital clubbing, pachydermia (thickening and wrinkling on the skin of the face and/or scalp), and periostosis (increase of periarticular tissue and subperiosteal neoformation bone). Other manifestations include coarse facial features, polyarthritis, cutis verticis gyrata (24%), seborrhea, palpebral ptosis, hyperhidrosis, acne, arthropathy, and acro-osteolysis of the long bones.^1^, ^2^

The generally recognized clinical presentations are as follows: the complete form, involving all three major symptoms; the incomplete form, with periostosis but without pachydermia; and the forme frustra with pachydermia and minimal or no skeletal anomalies. Both forms are autosomal dominant with incomplete penetrance and recessive inheritance has been suggested.^1^, ^2^, ^3^, ^4^ It begins during childhood or adolescence and progresses gradually over the next five to 20 years before stabilizing.^3^

The pathogenesis of PDP is still not clearly understood. The disease has been mapped to chromosome 4q33-q34 and mutations in HPGD, which encodes for 15-hydroxyprostaglandin dehydrogenase, the main enzyme of prostaglandin degradation, have been identified.^5^, ^6^

Increased levels of prostaglandin E2 (PGE2) resulting from defective degradation due to the implicated gene mutations, HPGD and SLCO2A1, appear to contribute to the pathogenesis of PDP. The severity of pachydermia and associated histological changes have been correlated with serum PGE2 levels and SLCO2A1 genotypes. PGE2 can mimic the activity of osteoblasts and osteoclasts, which may be responsible for the acro-osteolysis and periosteal bone formation. Moreover, the prolonged local vasodilatory effects of PGE2 may explain digital clubbing.^7^, ^8^

The reported case shows a complete form of the syndrome in a male patient with a history beginning in adolescence (typical epidemiology), with compatible clinical and radiological findings, presenting the three cardinal findings in addition to other associated manifestations, such as hyperhidrosis and acne. Although it is known that one-third of PDP patients have a family history,^3^ the present patient did not have relatives with suspected characteristics. A normal level of IGF-1 is strong evidence that the patient does not have acromegaly, which is an important differential diagnosis.^3^

A definitive treatment for disease has not been established. Symptomatic management with non-steroidal anti-inflammatory drugs (NSAIDs), simple analgesics, and intravenous bisphosphonates is currently being used. In addition, tamoxifen has been reported to be effective for arthralgia that is refractory to NSAIDs.^3^

The report in question recalls the importance of considering PDP as a possible diagnosis in dermatology and shows that establishing a diagnosis of PDP can be extremely challenging even for the more experienced physicians, mainly due to the rarity of the disease.

## Financial support

None declared.

## Authors’ contribution

Mônica Larissa Padilha Honório: Composition of the manuscript; intellectual participation in the propaedeutic and/or therapeutic conduct of the studied cased; critical review of the literature; critical review of the manuscript.

Guilherme Holanda Bezerra: Composition of the manuscript; critical review of the literature.

Vivianne Lira da Câmara Costa: Approval of the final version of the manuscript; participation in the study orientation; critical review of the manuscript.

## Conflicts of interest

None declared.

## References

[1] Sandoval A.R., Flores-Robles B.J., Llanos J.C., Porres S., Dardón J.D., Harrison R.M. (2013). Cutis verticis gyrata as a clinical manifestation of Touraine-Solente-Gole’ syndrome (pachydermoperiostosis). BMJ Case Rep.

[2] Lee S., Park S.Y., Kwon H.J., Lee C.H., Kim O.H., Rhee Y. (2016). Identification of the mutations in the prostaglandin transporter gene SLCO2A1 and clinical characterization in korean patients with pachydermoperiostosis. J Korean Med Sci.

[3] Abdullah N.R.A., Jason W.L.C., Nasruddin A.B. (2017). Pachydermoperiostosis: a rare mimicker of acromegaly. Endocrinol Diabetes Metab Case Rep.

[4] Karimova M.M., Halimova Z.Y., Urmanova Y.M., Korbonits M., Cranston T., Grossman A.B. (2017). Pachydermoperiostosis masquerading as acromegaly. J Endocr Soc.

[5] Uppal S., Diggle C.P., Carr I.M., Fishwick C.W., Ahmed M., Ibrahim G.H. (2008). Mutations in 15-hydroxyprostaglandin dehydrogenase cause primary hypertrophic osteoarthropathy. Nat Genet.

[6] Nakazawa S., Niizeki H., Matsuda M., Nakabayashi K., Seki A., Mori T. (2015). Involvement of prostaglandin E2 in the first Japanese case of pachydermoperiostosis with HPGD mutation and recalcitrant leg ulcer. J Dermatol Sci.

[7] Kim H.J., Koo K.Y., Shin D.Y., Kim D.Y., Lee J.S., Lee M.G. (2015). Complete form of pachydermoperiostosis with SLCO2A1 gene mutation in a Korean family. J Dermatol.

[8] Coggins K.G., Coffman T.M., Koller B.H. (2008). The Hippocratic finger points the blame at PGE2. Nat Genet.

